# METTL3-IGF2BP3-axis mediates the proliferation and migration of pancreatic cancer by regulating spermine synthase m6A modification

**DOI:** 10.3389/fonc.2022.962204

**Published:** 2022-10-06

**Authors:** Zhenyun Guo, Xiang Zhang, Chengjie Lin, Yue Huang, Yun Zhong, Hailing Guo, Zhou Zheng, Shangeng Weng

**Affiliations:** ^1^ Department of Hepatobiliary Surgery, The First Affiliated Hospital of Fujian Medical University, Fuzhou, China; ^2^ Fujian Abdominal Surgery Research Institute, The First Affiliated Hospital, Fujian Medical University, Fuzhou, China; ^3^ Key Laboratory of Gastrointestinal Cancer (Fujian Medical University), Ministry of Education, Fuzhou, China; ^4^ Fujian Key Laboratory of Tumor Microbiology, Department of Medical Microbiology, Fujian Medical University, Fuzhou, China

**Keywords:** spermine synthase, polyamine, m6A, pancreatic cancer, EMT, AKT

## Abstract

Spermine synthase (*SMS*) is an enzyme participating in polyamine synthesis; however, its function and role in pancreatic cancer remains elusive. Here we report that *SMS* is upregulated in pancreatic cancer and predicts a worse overall survival and significantly promotes the proliferation and migration of pancreatic cancer cells. Excessive *SMS* reduces the accumulation of spermidine by converting spermidine into spermine, which activates the phosphorylation of serine/threonine kinase (AKT) and epithelial-mesenchymal transition (EMT) signaling pathway, thereby inhibiting pancreatic cancer cell proliferation and invasion. Moreover, *SMS* was identified as the direct target of both methyltransferase like 3 (*METTL3*) and insulin like growth factor 2 mRNA binding protein 3 (*IGF2BP3*), which directly bind to the m6A modification sites of *SMS* and inhibit mRNA degradation. Knockdown of *METTL3* or *IGF2BP3* significantly reduced the *SMS* protein expression and inhibited the migration of pancreatic cancer. We propose a novel regulatory mechanism in which the METTL3-IGF2BP3 axis mediates the mRNA degradation of SMS in an m6A-dependent manner to regulate spermine/spermidine conversion, which regulates AKT phosphorylation and EMT activation, thereby inducing tumor progression and migration in pancreatic cancer.

## Introduction

Pancreatic cancer is a highly malignant tumor with the highest annual mortality among all cancers (1). At present, the main treatment for pancreatic cancer is surgical removal (2); however, most pancreatic cancers have already metastasized at the time of diagnosis (3). The molecular mechanism of pancreatic cancer metastasis has not been clarified. Thus, it is important to uncover the mechanism of how pancreatic cancer metastasizes to provide potential therapeutic targets for more effective management of this deadly disease.

m6A is the most abundant epigenetic modification in eukaryotic mRNAs and is tightly and closely correlated to many fundamental biological processes (4) (5). Numerous studies have found that m^6^A modification plays an important role in the occurrence and development of tumors including pancreatic cancer (4, 6, 7) (8–10). Xia et al. found that *METTL3* can promote the proliferation and invasion of pancreatic cancer (9). Y. Chen found that *ALKBH5* can mediate *PER1* m6A and then regulate the EMT of pancreatic cancer (11). It has also been suggested that *METTL3* inhibits tumorigenesis (12). In view of the multiple roles of m^6^A in pancreatic cancer, more in-depth research is needed.

*SMS* is an enzyme involved in the synthesis of spermine, which converts spermidine into spermine and plays an important role in maintaining the homeostatic balance of polyamines in the cell (13). The native polyamines include putrescine, spermidine and spermine, which are small-molecule polar compounds containing two or more amino groups (14). Polyamines can regulate metabolism, intracellular DNA oxidative damage stress (15), and are important factors for cell growth and development. Studies have reported that polyamines are elevated to varying degrees in most tumor cells. Moreover, polyamines can promote the progression of pancreatic cancer (16). However, the effects of the changes in various components of polyamines are rarely studied in the context of pancreatic cancer. Studies have shown that extremely high levels of spermidine can inhibit the growth of myeloma (17). In addition, spermine plays an important role as an immune suppressant. Studies have shown that spermidine can affect the NADPH oxidase activation of neutrophils and thus inhibit the immune response (18, 19). Some researchers believe that exogenous spermidine can inhibit the phosphorylation of AKT causing inactivation, thereby promoting autophagy and ultimately leading to cell growth (20). Spermine synthetase can alter the ratio of polyamine components in the cell by synthesizing spermidine into spermine (21). Therefore, it is worthwhile to examine the role of spermine synthase in pancreatic cancer.

EMT is an important sign of tumor migration and invasion (22). As an important signal transduction pathway, the PI3K-AKT pathway plays a key role in tumor proliferation and migration (23). PI3K-AKT pathway can regulate the EMT pathway, leading to tumor cell migration and invasion (24). We speculate that a reduced spermidine to spermine ratio further leads to weakened inhibition of AKT phosphorylation. The phosphorylated AKT can promote the EMT transition of cells through the signal amplification mechanism, and ultimately lead to tumor metastasis.

However, there are only a few reports describing the role of SMS in pancreatic cancer (18, 25). In this study, we explored the clinical relationship between SMS and pancreatic cancer, investigated its biological functions and the molecular mechanism by which SMS promotes the progression of pancreatic cancer.

## Materials and methods

### Patients and specimens

Clinical tissue samples from 59 patients were obtained from The First Affiliated Hospital of Fujian Medical University (Fuzhou, China). All experiments involving human samples and clinical data were approved by the Accreditation Committee of The First Affiliated Hospital of Fujian Medical University.

### Public datasets

The public datasets used in this study included four GEO datasets (http://www.ncbi.nlm.nih.gov/geo/, GSE15471, GSE16515, GSE71989 and GSE22780). GEPIA (http://gepia.cancer-pku.cn/),the m6A-ATLAS database (www.xjtlu.edu.cn/biologicalsciences/atlas).

### Immunohistochemistry

Immunohistochemistry staining was performed using antibodies targeting SMS (Novus, 1:800), METTL3 (Abcam, 1:2000), IGF2BP3 (Abcam, 1:200), AKT (CST, 1:200), p-AKT(T308) (CST, 1:200) and p-AKT(S473) (CST, 1:200). The immunoreactivity was scored blindly according to the value of immunoreaction intensity (none = 0; weak = 1; intermediate = 2; and strong = 3) and the percentage of tumor cell stained (none = 0; <10% = 1; 10–50% = 2; >50% = 3). The intensity and percentage values were added to provide immunoreactivity score ranging from 0 to 6. High expression of SMS was defined as an immunohistochemical score of ≥5, and low expression was defined as <5. (26)

### Establishment of cell lines

Human pancreatic cancer cell lines ASPC-1, PANC-1, BxPC-3, SW1990, Mia-Paca2 were purchased from American Type Culture Collection (ATCC, USA). Cells were cultured in Dulbecco’s Modified Eagle’s medium (DMEM) supplemented with 2 mM L-glutamine and 10% FBS. Cells were cultured in a 37 °C, 5% CO_2_ incubator. Plasmids were transfected in HEK-293 T cells using Lipofectamine 3.0 (Invitrogen) following the manufacturer’s instructions. Next, the pancreatic cancer cell lines with stable gene expression were selected in culture medium supplemented with puromycin (1 μg/ml; Sigma Aldrich). Small interfering RNA (siRNA) targeting METTL3/IGF2BP3 specific regions were synthesized by Gene Pharma (Shanghai, China). Transfections were carried out using Lipofectamine 3000 (Invitrogen) following the manufacturer’s instructions. The siRNA sequence is summarized in **Table S2**.

### Western blot analysis

For Western blot analysis, the nuclear and total cellular protein fractions were extracted with Western IP Lysis Buffer (Beyotime, China). The primary antibodies were incubated at 4^o^C overnight and then incubated with secondary antibodies (1:2000, CST) for 1h. Finally, they were washed using 1% TBST and detected by a chemiluminescence system.

### Colony formation assay

Cells were seeded into 6-well plates at a density of 500 cells/well and the culture was replaced with new medium every 48 hours, then were cultured for 10-14 days. The colonies were fixed in 4% paraformaldehyde and stained with 0.1% crystal violet (Beyotime, China). Numbers of colonies in triplicate wells were counted for each treatment group.

### Cell proliferation assay

1,000 cells suspended in 100 µL DMEM medium were seeded into 96-well plate. The cell proliferation was assessed by the CCK8 (Dojindo, Japan). 10 ul CCK8 solution CCK8 solution (10 µL) was added to each well of the plate after different incubation times. The absorbance was measured 2h later at 450 nm using a microplate reader after 2 hours.

### Cell invasion assays

Cell invasion was assessed with transwell plates (BD Biosciences, USA) as previously described (27).

### Animal experiments

Male BALB/c nude mice (5-week old) were purchased from Beijing Vital River Laboratory Animal Technology Co, Ltd. (Beijing, China), and cultured at the specific pathogen-free (SPF) facility. A total of 3× 10^6^ stably transfected cells with genetically altered expression of SMS were subcutaneously injected into the axillary fossa of the nude mice (eight mice per group). Tumor length (L) and width (W) were measured every 4 days, and tumor volume was calculated as 0.5×L×W^2^. Stably transfected Mia-Paca2 cells with SMS overexpression were then injected into the tail veins of nude mice at a dose of 5×10^6^ cells/mouse to establish the lung metastasis model.

### Measurement of intracellular polyamines

Chromatography was performed with a Shiseido nanospace SI-2 HPLC system (Shiseido Co., Tokyo, Japan) coupled to a Shiseido MG C18 column (5 μm, 150 × 1.5 mm i.d.). A gradient eluent (A, 0.2% acetic acid; B, 0.2% acetic acid in acetonitrile) at100 μL/min was used. All data were recorded on a Thermo LCQ advantage iontrap MS equipped with electrospray ionization (ESI; Thermo,San Jose, CA, USA) operated in the positive ionization mode. The operating conditions were set as follows: spray voltage, 6 kV; capillary voltage, 4 V; tube lens offset voltage,40 V; sheath gas flow rate, 30 units; and capillary temperature, 250°C. In tandem MS analysis, the protonated molecular ions were fragmented by helium gas collisions. Levels of polyamines were normalized to protein amount (28).

### RNA extraction, reverse transcription and quantitative PCR

Total RNA was extracted by Trizol Reagent (Invitrogen) from cells. cDNA was obtained from total RNA with PrimeScript™ RT reagent kit (Takara Bio, Inc., Otsu, Japan). The mRNA expression was assessed by Real-time quantitative PCR. The primers for RT-qPCR are shown in **Table S3**.

### mRNA stability assay

Cells were plated in 6-wells dish and incubated with actinomycin D (Santa Cruz) at 5 µM for the indicated time. The first time point (t = 0 h) was taken after 10 min, then 4 and 8 h. Total RNA extracted from each sample was used for reverse transcription and qRT–PCR analysis.

### MeRIP-qPCR

M^6^A RNA immunoprecipitation (MeRIP) was performed with Magna MeRIP m^6^A kit (17–10, 499, Millipore) according to the manufacturer’s instructions.

### Luciferase reporter assay

The wild type (SMS-WT) and m^6^A sites mutated SMS (SMS-MUTE 1, 2, 3) were constructed into luciferase reporter vector pmir-GLO. After 48 h transfection, the cells were lysed by passive lysis buffer. Firefly Luciferase and Renilla Luciferase of lysis were detected, respectively.

### Statistical analysis

Statistical analyses were performed using the SPSS software (version 17.0). Differences between the indicated groups were compared using the t-tests and one-way analysis of variance (ANOVA) followed by Fisher’s least significant difference (LSD) test. The cumulative overall survival (OS) rates were calculated using the Kaplan–Meier method, and differences between curves were evaluated using the log-rank test. P value < 0.05 was considered statistically significant.

## Results

### *SMS* expression is increased in pancreatic cancer and is related to prognosis

**Figure 1:**
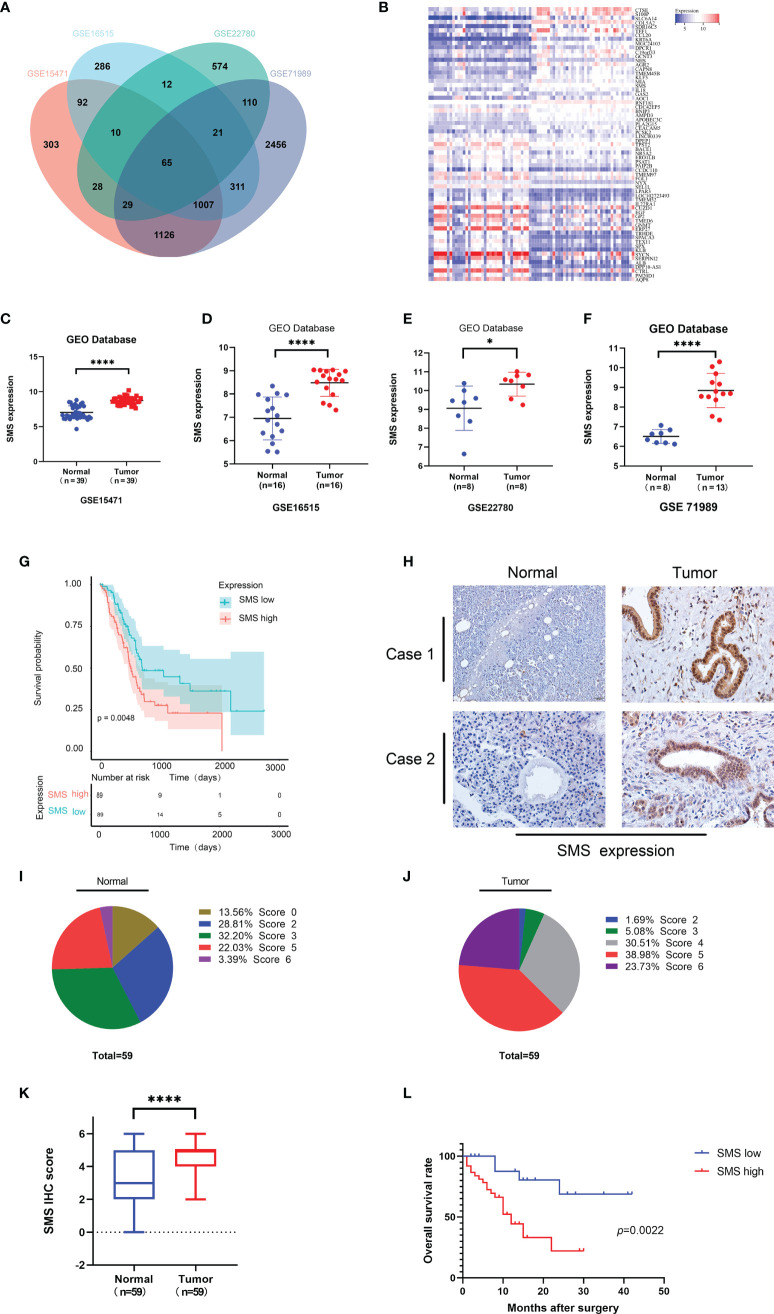
The expression and prognostic value of SMS in human pancreatic cancer. **(A)** Coincidence of differential Genes in GSE15471, GSE16515, GSE22780, and GSE71989 from GEO database. **(B)** The heat map of up-regulated genes and down-regulated genes in overlapping genes. **(C-F)** Expression level of SMS in pancreatic tumor and adjacent normal tissues from GEO database were analyzed. **(G)** Kaplan–Meier analyses of SMS high expression and low expression were analyzed from the TCGA database. **(H-K)** The expression of SMS in 59 paraffin embedded specimens from the internal cohort was determined by IHC staining. Representative IHC images are shown **(H)**, and the relative SMS staining intensity was scored **(I-K)**. Scale bar, 200 μm. **(L)** Kaplan–Meier analyses of the correlations between SMS expression and overall survival of all PDAC patients. *P < 0.05; ****P < 0.0001.

We analyzed the GEO database to determine the differentially expressed genes in pancreatic cancer and took the intersection of differential genes in GSE15471, GSE16515, GSE22780, and GSE71989. The results showed that there were 65 overlapping genes (**Figure 1A**), with 46.15% up-regulated and 53.85% down-regulated (**Figure 1**). Among these genes, *SMS* was the only gene related to polyamine metabolism. We further analyzed the expression of *SMS* in pancreatic cancer tissues. By analyzing the results in the GEO database, we found that the expression of *SMS* in pancreatic cancer tissues was significantly higher than that in adjacent tissues (**Figures 1C–F**). Additionally, we analyzed The Cancer Genome Atlas (TCGA) database and the results showed that the expression of SMS in cancer tissue specimens was significantly higher than that in adjacent tissues, and the expression of SMS was related to the prognosis of pancreatic cancer. The survival time of patients with high *SMS* expression was much shorter than that of the low expression group after surgery (P < 0.05) (**Figure 1G**). To further verify the results of bioinformatics analysis, we performed immunohistochemical analysis on 59 cases of cancerous and adjacent tissues. The results showed that the expression of *SMS* in cancerous tissues was significantly higher than that in adjacent tissues (**Figures 1H–K**). Moreover, survival analysis showed that the postoperative survival time of the SMS high expression group was significantly shorter than that of the low expression group (**Figure 1L**). Based on clinical-pathological data of patients, chi-square test analysis showed that the expression level of *SMS* was correlated with lymph node metastasis and tumor stage of pancreatic cancer, but not with sex, age, tumor differentiation, and tumor size (**Table S1**). Therefore, the expression of *SMS* in pancreatic cancer is increased and represents an important factor in the progression of pancreatic cancer, which is in line with the findings of Phanstiel et al in their study the relative mRNA expression of SMS was significantly increased in both PanIN and PDAC samples of 223 human patients (25).

### *SMS* can promote the growth, migration, and invasion of pancreatic cancer *in vitro*

**Figure 2:**
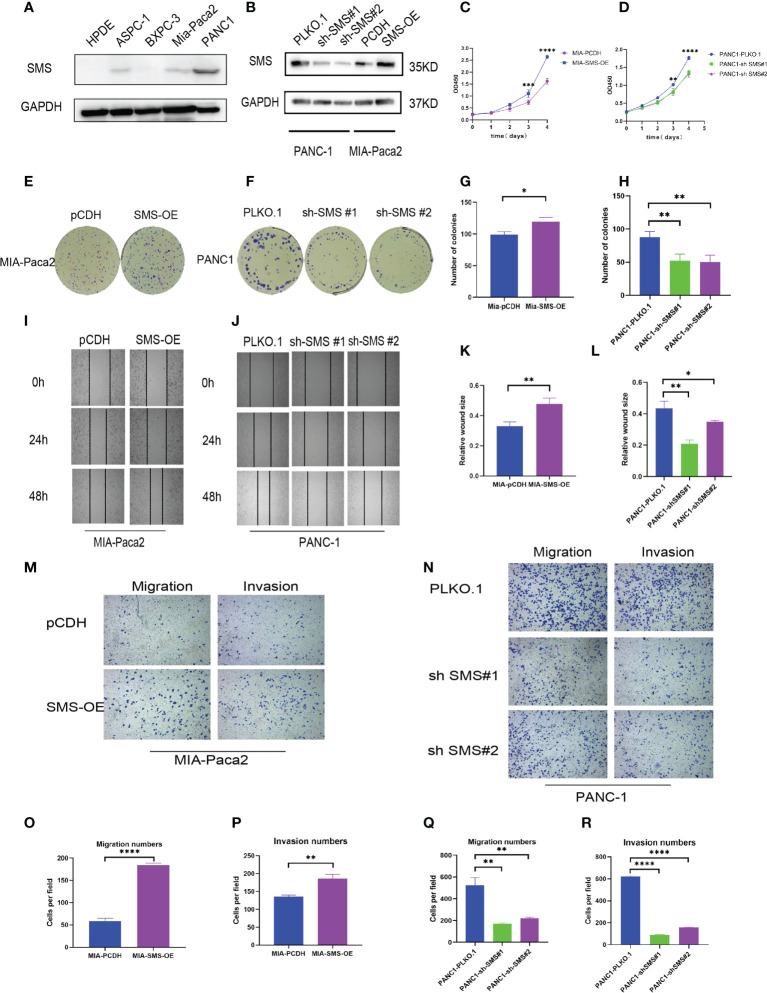
*SMS* can promote the growth, migration and invasion of pancreatic cancer. **(A)** The protein levels of *SMS* in normal human pancreatic duct epithelial (HPDE) cells and selected human pancreatic cancer cell lines were quantitated by western blot assays. **(B)** Mia-Paca2 cells with stable SMS overexpression PCDH-SMS-OE and PANC-1 cells with *SMS* knockdown PLKO.1-shSMS#1 and PLKO.1-shSMS#2 were generated. The changes in *SMS* expression were conformed using western blot. **(C, D)** The proliferative ability of stably transfected PANC-1 or Mia-Paca2 cells was investigated *via* CCK-8 assays. **(E-H)** Representative colony formation images are shown **(E, F)**, and the numbers of colonies were summarized **(G, H)**. **(I-L)** Wound-healing assays with stably transfected Mia-Paca2 **(I)** and PANC-1 **(J)** cells were performed. Representative images and quantifcation of wound closure are presented **(K, L)**. **(M-R)** Transwell assays with stably transfected Mia-Paca2 **(M)** and PANC-1 **(N)** cells were performed. Representative images and quantifcation of the results are presented. **(O-R)** *P < 0.05; **P < 0.01; ***P < 0.001; ****P < 0.0001.

Western blotting showed that compared to the immortalized pancreatic epithelial cells HPDE, *SMS* expression in other cell lines was increased to varying degrees, and PANC-1 was representative of high expression, while Mia-Paca2 expression increased slightly (**Figure 2A**). Therefore, we used lentiviral transfection technology to construct a stable transfected cell line (**Figure 2B**). The growth curve showed that over-expression of *SMS* can promote the growth of pancreatic cancer cells, while knockdown of *SMS* can inhibit their proliferation (**Figure 2C**). Colony formation experiments also showed that increasing the level of *SMS* expression can increase the number of clones, whereas reducing *SMS* expression can inhibit the formation of clones (**Figures 2E–H**).The results of the wound-healing experiment showed that over-expression of *SMS* can promote the migration of pancreatic cancer cells while knocking down *SMS* significantly inhibited the migration rate (**Figures 2I–L**). Additionally, the results of transwell assays demonstrated that overexpression of *SMS* can promote the migration and invasion of pancreatic cancer cells, while knockdown of *SMS* had the opposite effect (**Figures 2M–R**). Therefore, increased expression of *SMS* in pancreatic cancer cells promotes the proliferation, migration, and invasion of pancreatic cancer cells.

### *SMS* promotes pancreatic cancer metastasis, which is mediated through the AKT/EMT signaling pathway

**Figure 3:**
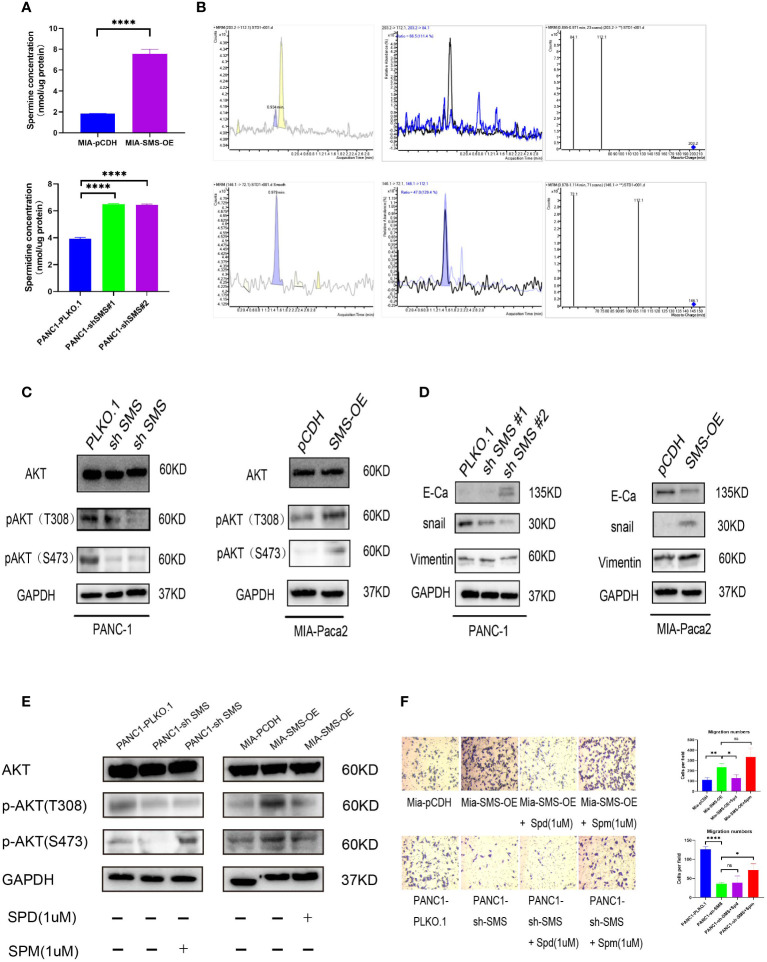
SMS promotes pancreatic cancer metastasis is mediated through the AKT/EMT signaling pathway. **(A)** SMS metabolizes spermidine to spermine. The levels of spermidine (SPD), and spermine (SPM) in SMS-OE or SMS-knockdown Mia-Paca2 and PANC1 cells were determined by LC-MS. **(B)** Mass spectrometry images of spermidine and spermine detection. **(C, D)** The protein expressions of AKT and EMT pathway were analyzed. **(E)** Spermidine/spermine could rescue the expression of AKT phosphorylation. **(F)** Spermidine/spermine could rescue the migration ability of overexpressed/knockdown SMS-stable cells by transwell assay. *P < 0.05; **P < 0.01; ****P < 0.0001. ns, no significance.

Treatment with spermidine *in vitro* can inhibit the phosphorylation of AKT, thereby inhibiting cell growth (20). We speculate that changes in the expression of SMS in pancreatic cancer could affect the levels of spermidine and spermine in cells, while changes in spermidine/spermine levels could affect the phosphorylation of AKT and ultimately affect the activation and inhibition of the AKT signaling pathway. Therefore, we detected spermine and spermidine levels in the cells. The results showed that the spermine level was significantly increased in cells overexpressing SMS, while after knocking down SMS, the spermidine level was significantly accumulated (**Figures 3A, B**). It is noteworthy that similar results of spermidine buildup have been seen in the fibroblasts of patients with Snyder Robinson Syndrome which have defective SMS (29). To further verify the relationship between *SMS* and *AKT*, western blot analysis was performed and showed that over-expression of *SMS* can up-regulate the level of p-AKT while knocking down *SMS* can reduce the expression of p-AKT (**Figure 3C**). We further verified the relationship between *SMS* content and the corresponding pathway. The results showed that *SMS* overexpression can promote the EMT pathway. As a key protein in EMT, E-cadherin was decreased whereas snail and Vimentin were increased. Inhibition of *SMS* expression had the opposite effect (**Figure 3D**). Moreover, we have also found that overexpression or knockdown of *SMS* expression can affect *ERK* and *mTOR* phosphorylation (**Figures S2 A, B**). Furthermore, we found that the addition of spermidine/spermine could reverse the expression of AKT phosphorylation (**Figure 3E**) and the migratory ability of *SMS*-overexpressed or knockdown -stable cells by Transwell assay (**Figure 3F**). Therefore, *SMS* promotes AKT phosphorylation and affects the EMT pathway by converting spermidine to spermine, ultimately leading to pancreatic cancer progression. It should be realized that SMS may also directly act on other oncogenes through protein-protein interactions. There are three domains in SMS protein (29, 30). By searching The Molecular INTeraction Database (MINT database), we found some proteins that may interact with SMS proteins, including IMMP2L, MAPK6, MAPKAPK3, MAPK8IP2, RPS6KA3. Furthermore, through Co-IP experiments we found that SMS protein may interact with MAPKAPK3 (data not shown), but this part of the mechanism needs to be verified in future studies.

### *METTL3* and *IGF2BP3* are involved in m6A modification of *SMS* and promote pancreatic cancer progression

**Figure 4:**
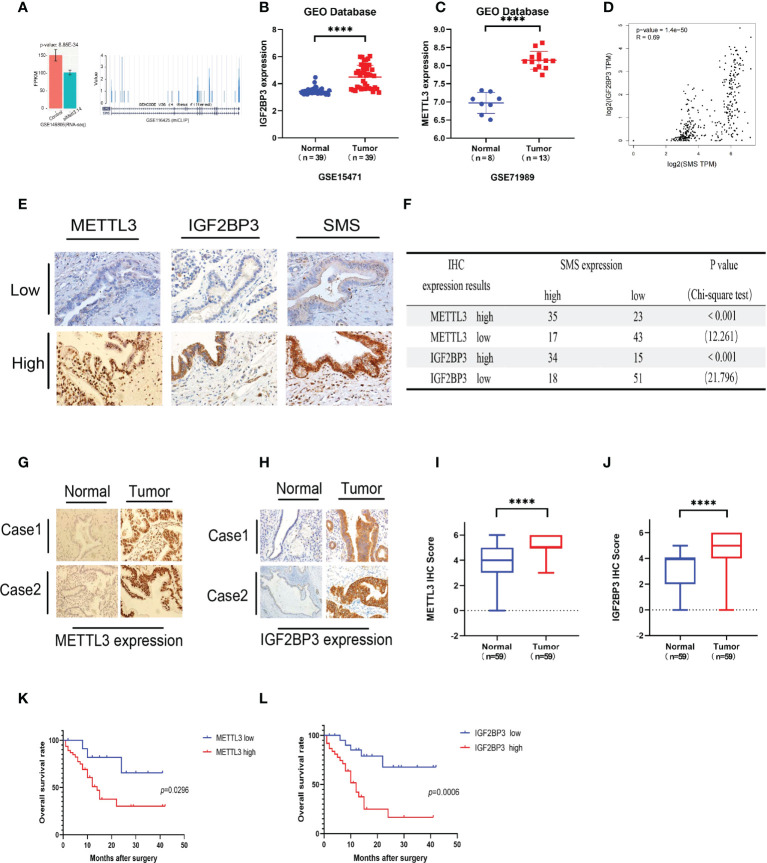
METTL3 and IGF2BP3 are highly expressed in pancreatic cancer and predicts poor prognosis of pancreatic cancer patients. **(A)** The expression of SMS in the METTL3 and METL14 double knock chips in the GEO database. **(B, C)** The expression of METTL3 and IGF2BP3 in cancer tissues and adjacent tissues. **(D)** The GEPIA database showed that IGF2BP3 was positively correlated with SMS mRNA expression. **(E)** The expressions of METTL3/IGF2BP3 and SMS in tissues of PDAC patients were evaluated by IHC. **(F)** The correlation between METTL3/IGF2BP3 and SMS levels in tissues was analyzed. **(G-J)** The expression of METTL3 and IGF2BP3 in pancreatic cancer tissues was higher than that in adjacent tissues by IHC, and the relative METTL3/IGF2BP3 staining intensity was scored. **(K, L)** Kaplan–Meier analyses of the correlations of METTL3/IGF2BP3 expressions and overall survival of PDAC patients. ****P < 0.0001.

We have demonstrated that expression of *SMS* is upregulated in pancreatic cancer, thereby promoting its progression. However, the underlying mechanism remains to be determined. By searching the GEO database, we found that knockout of both *METTL3* and *METTL4* significantly reduced the expression of *SMS* (**Figure 4A**). Through the GEO database, it was found that expression of *METTL3* and *IGF2BP3* in pancreatic cancer tissues were higher than those in adjacent tissues (**Figures 4B, C**). We also analyzed the expression of m6A readers in pancreatic cancer tissues from the TCGA database, and the expression of *IGF2BP3* was associated with the prognosis of pancreatic cancer (**Figure S1A**). Additionally, the GEPIA online prediction website showed that the expression of *IGF2BP3* mRNA in pancreatic cancer was positively correlated with the expression of *SMS* (**Figure 4D**). Therefore, we speculate that *METTL3* can regulate *SMS* mRNA m6A methylation and be recognized by *IGF2BP3* to further affect the prognosis of pancreatic cancer. Furthermore, the results of the immunohistochemical analysis showed that the expression of *METTL3* and *IGF2BP3* was consistent with that of *SMS* (**Figure 4E**). All results were scored and a chi-square test was performed, with the results showing that the expression of *METTL3/IGF2BP3* was significantly correlated with *SMS* expression (**Figure 4F**). These findings indicate that METTL3 may play a regulatory role in *SMS* expression. Moreover, it was found that the expression levels of *METTL3* and *IGF2BP3* were higher in cancer tissues than those in adjacent tissues (**Figures 4G–J**). Survival analysis showed that the expression of *METTL3* and *IGF2BP3* affected the prognosis of pancreatic cancer patients and that high expression of *METTLE/IGF2BP3* can promote the progression of pancreatic cancer (**Figures 4K, L**). Therefore, *METTL3* and *IGF2BP3* may play key roles in the m6A modification of SMS and pancreatic cancer progression.

### *METTL3* and *IGF2BP3* can promote the migration and invasion of pancreatic cancer

**Figure 5:**
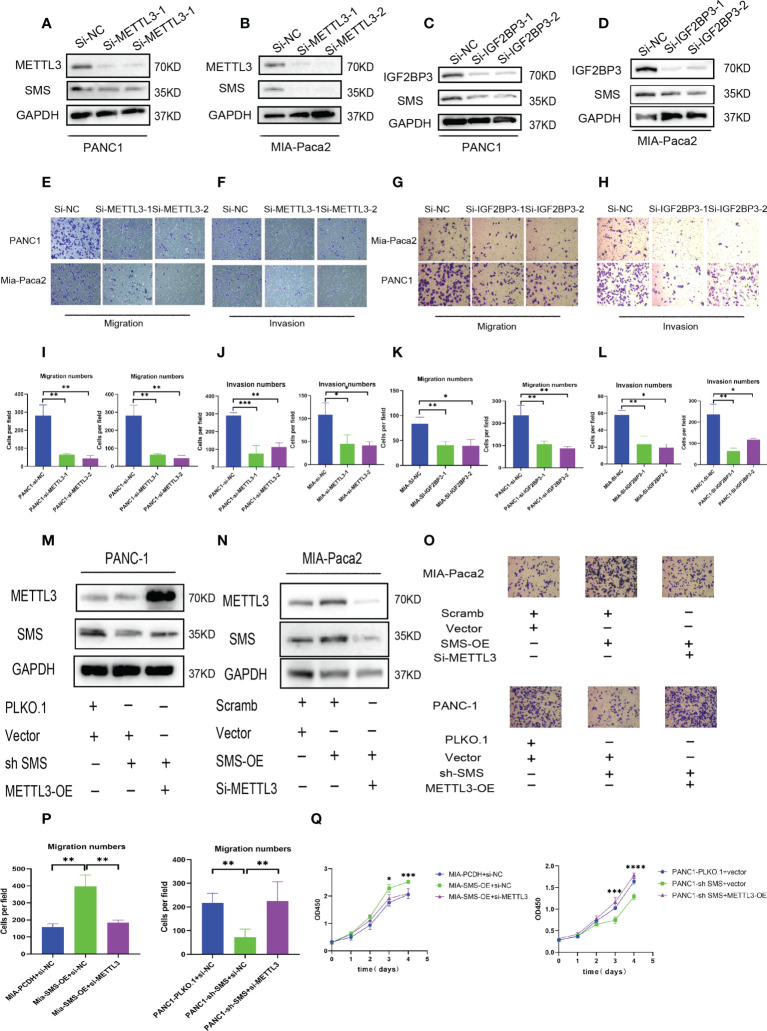
*METTL3* and *IGF2BP3* regulate *SMS* expression and promote pancreatic cancer metastasis. **(A-D)** The expression level of *SMS* interferes with the expression of *METTL3* and *IGF2BP3* in Mia-Paca2 and PANC1 cells. **(E-L)** Transwell assays of SMS interferes with the expression of *METTL3* and *IGF2BP3* in Mia-Paca2 and PANC1 cells were performed. Representative images and quantifcation of the results are presented. **(M, N)** *METTL3* reversed the expression of *SMS* in stable SMS overexpression/knockdown PDAC cell. **(O, P)** Transwell experiments found that *METTL3* could reverse the migration ability of stable SMS overexpression/knockdown PDAC cell. **(Q)** CCK-8 experiments found that *METTL3* could reverse the proliferation of stable SMS overexpression/knockdown PDAC cell. *P < 0.05; **P < 0.01; ***P < 0.001; ****P < 0.0001.

**Figure 6:**
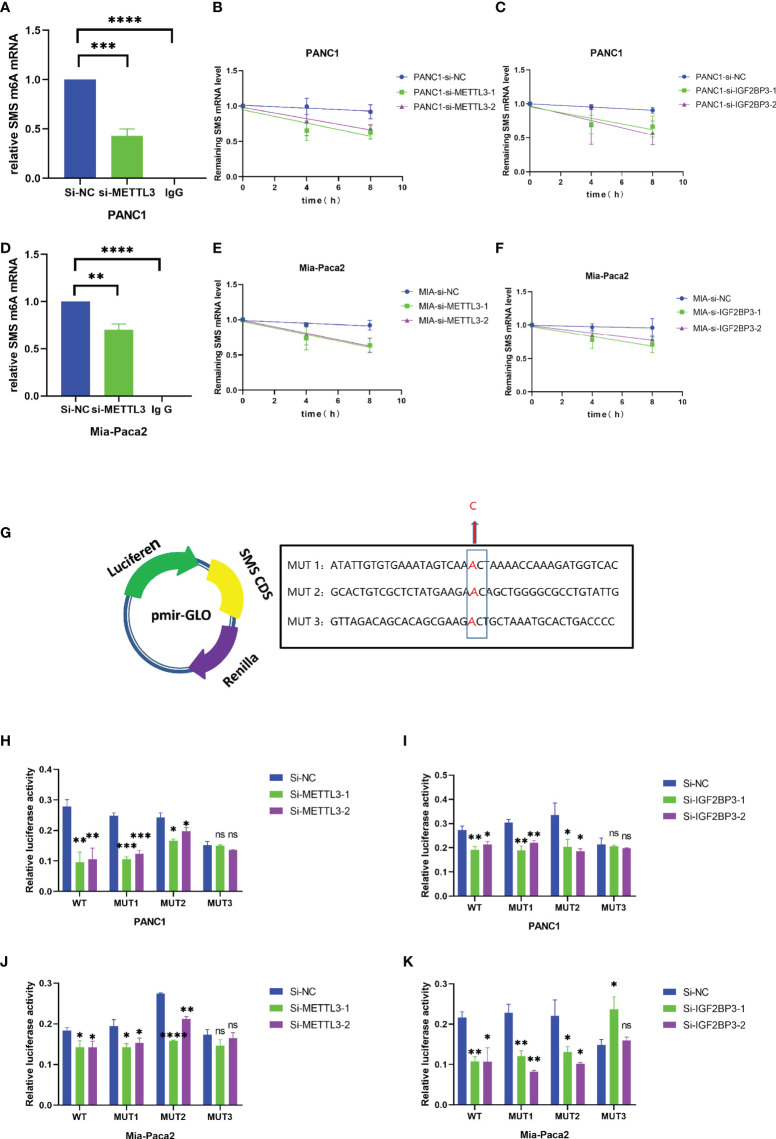
The METTL3-IGF2BP3 axis promotes increased *SMS* mRNA stability and protein expression by mediating *SMS* m6A modification. **(A, D)** ME-RIP assays showed the relative percentage of *SMS* mRNA with methylation. **(B, C, E** and **F)** The mRNA stability and degradation halftime of *SMS* in Mia-Paca2 and PANC1 treated by Actinomycin D. **(G)** Wild-type or mutant 1-3 sites m6A consensus sequence was fused with firefly luciferase reporter, respectively. **(H-K)** Mutation of m6A sites in *SMS* (constructed in firefly reporter) repressed the luciferase expression of reporter. *P < 0.05; **P < 0.01; ***P < 0.001; ****P < 0.0001. ns, no significance.

To further explore the role of *METTL3* and *IGF2BP3* in pancreatic cancer, we conducted western blot experiments by transfecting METTL3-siRNA and IGF2BP3-siRNA into PANC-1 and Mia-Paca2. The results showed that compared to the control group, interference with *METTL3* and *IGF2BP3* expression can reduce the expression of *SMS* (**Figures 5A–D**). Therefore, we further explored the role of *METTL3* and *IGF2BP3* on pancreatic cancer cell. In the migration and invasion experiments, interfering with the expression of *METTL3* and *IGF2BP3* inhibited the migration and invasion of pancreatic cancer (**Figures 5E–L**). Moreover, wound healing assay results showed that knockdown of *METTL3* expression significantly inhibited cell migration (**Figure S1B**). Furthermore, the overexpression of *METTL3* reversed the expression of *SMS* in *SMS* stable knockdown cells, while knockdown of *METTL3* reversed the protein expression of *SMS* in overexpressed *SMS* (**Figures 6M, N**). The Transwell migration assay also demonstrated that *METTL3* could reverse the migration ability of overexpressed/knockdown SMS pancreatic cancer cells (**Figures 5O, P**). The results of the CCK-8 assay showed that *METTL3* could reverse the proliferation of stably transfected cells (**Figure 5Q**). Therefore, the highly expressed SMS protein in pancreatic cancer may be related to the expression of *METTL3* and *IGF2BP3*, and the expression of *METTL3* and *IGF2BP3* related to the migration and invasion of pancreatic cancer.

### The METTL3-IGF2BP3 axis promotes increased *SMS* mRNA stability and protein expression by mediating *SMS* m6A modification

We next performed a methylated RNA immunoprecipitation (ME-RIP) analysis to further explore the regulatory relationship between the METTL3-IGF2BP3-axis and *SMS*. The results showed that knockdown of METTL3 significantly reduced m6A level of SMS mRNA (**Figures 6A, D**). The RNA stability experiment results showed that compared to the control group, interference with *METTL3* and *IGF2BP3* led to an increase in the degradation rate of *SMS* mRNA (**Figures 6B, C, E, F**). We then constructed three mutant SMS CDS plasmids for the luciferase reporter assay to determine the specific modification sites (**Figure 6G**). The results of the dual-luciferase reporter experiment showed that the transcriptional level of wild-type, MUT1 or MUT2, but not MUT3, was significantly decreased with METTL3 or IGF2BP3 knockdown (**Figures 6H–K**), assuming that the regulation of SMS expression was under control of METTL3 associated m6A modification, which was acted mainly through the MUT3 site since SMS expression in the presence of intact Site 3 as in wild-type, MUT1 or MUT2 correlated with METTL3 status.

### *SMS* can promote tumor growth and metastasis *in vivo*

**Figure 7:**
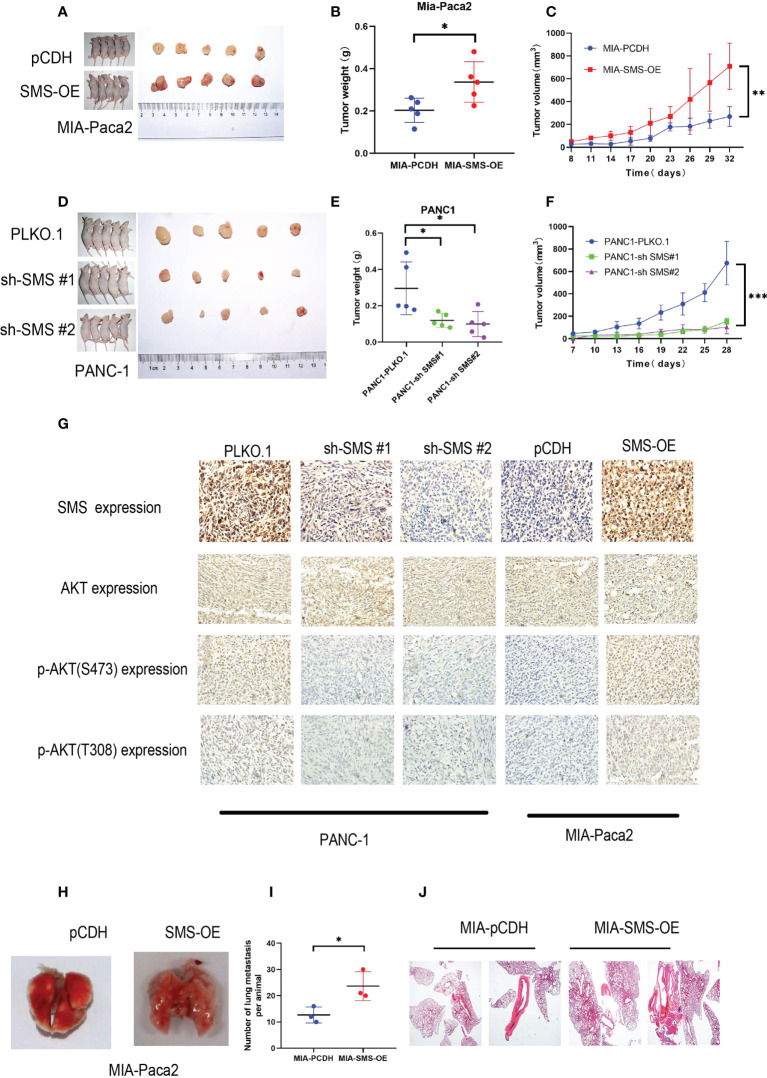
*SMS* can promote tumor growth and metastasis *in vivo*. **(A, D)** Tumor xenograft models were constructed with stable SMS-overexpressing (n = 5) or SMS-knockdown (n = 5) Mia-Paca2 cells and PANC1 corresponding negative control cells. **(B, E)** Then tumors were collected from sacrificed mice and tumor weights were measured. **(C, F)** The size of the tumors was measured at the indicated time points. **(G)** Tumors from mice were analyzed by IHC staining, Scale bar, 200 µm. **(H-J)** Representative images of lung metastasis and hematoxylin and eosin staining are shown. Metastatic nodules were counted with or without a microscope and recorded. Spd, spermidine;Spm, spermine.*P < 0.05; **P < 0.01; ***P < 0.001.

A subcutaneous tumor model was established by subcutaneously injecting the pancreatic cancer cells into nude mice. Compared to the control group, overexpression of *SMS* can promote tumor growth, while knockdown of *SMS* can significantly inhibit tumor formation and slow the rate of tumor growth (**Figure 7A–F**). The immunohistochemistry results showed that knockdown of *SMS* resulted in decreased phosphorylation of AKT, while overexpression of *SMS* resulted in increased phosphorylation of AKT (**Figure 7**). Stably transfected Mia-Paca2 cells with SMS overexpression were then injected into the tail veins of nude mice to establish the lung metastasis model. Additionally, the anatomy of the lung metastasis model mice showed that the number of lung metastases in the *SMS* overexpression group was greater than that in the control group (**Figure 7I**). H-E staining showed similar results (**Figure 7**). Therefore, these results indicate that SMS can promote the growth and metastasis of pancreatic cancer *in vivo*.

## Discussion

At present, the incidence of pancreatic cancer is increasing annually (31). As a grave disease with high mortality, research into pancreatic cancer has achieved widespread attention. (1, 32) Despite extensive research, the specific molecular mechanism underlying the metastasis of pancreatic cancer has not yet been elucidated.

*SMS* is an enzyme in the pathway of polyamine synthesis, which can specifically convert spermidine to spermine and plays an important role in polyamine synthesis and metabolism (13). At present, there are relatively few studies on *SMS* in tumors, although studies have shown that overexpression of *SMS* can promote the progression of colon cancer (21). Ja Ladanki et al. demonstrated that the use of *SMS* inhibitors can inhibit the level of polyamines, thereby inhibiting tumor cell proliferation (33). Numerous studies have shown that the level of polyamines in tumors is significantly increased and that polyamines play a critical role in the growth of tumor cells (13, 16, 34). However, there remains little research on the role of each component in polyamines. Additionally, studies have shown that spermidine can inhibit the proliferation of myeloma cells, and exogenous spermidine can inhibit cell proliferation; this effect is caused by inhibiting the phosphorylation process of AKT (35). In this study, we confirmed that overexpression of *SMS* can promote the increase of spermine levels in pancreatic cancer cells, knockdown of SMS can lead to increased spermidine, and the level of *SMS* protein is positively correlated with AKT phosphorylation and the PI3K-AKT/EMT pathways. Moreover, at both cellular and animal levels, we also verified that SMS can promote the proliferation, migration, and invasion of pancreatic cancer.

The development of epigenetics has led to a deeper understanding of gene expression regulation (36, 37). m6A modification has been extensively studied as an important regulatory mechanism (38, 39). At present, the role of m6A in tumorigenesis and development has been proven (11, 40; Wei 38, 41). The role of m6A in pancreatic cancer has also been extensively studied, and some studies have shown that it can regulate pancreatic cancer progression by affecting alternative splicing of *METTL14* (42). In view of the multiple effects of m6A modification on tumors, further research is needed. By searching the m6A-ATLAs database, we found that *SMS* mRNA has many m6A modifications, with three m6A-specific sites predicted in *SMS* mRNA. Therefore, m6A modification plays a key role in the regulation of SMS expression. Through further exploration, we found that *METTL3* and *IGF2BP3* can significantly inhibit the migration and invasion of pancreatic cancer cells. Additionally, interference with the expression of *METTL3* and *IGF2BP3* increased the degradation rate of *SMS* mRNA. These results well explain why the expression of *SMS* in pancreatic cancer is elevated, as well as the main cause of polyamine anabolic disorder in pancreatic cancer.

**Figure 8:**
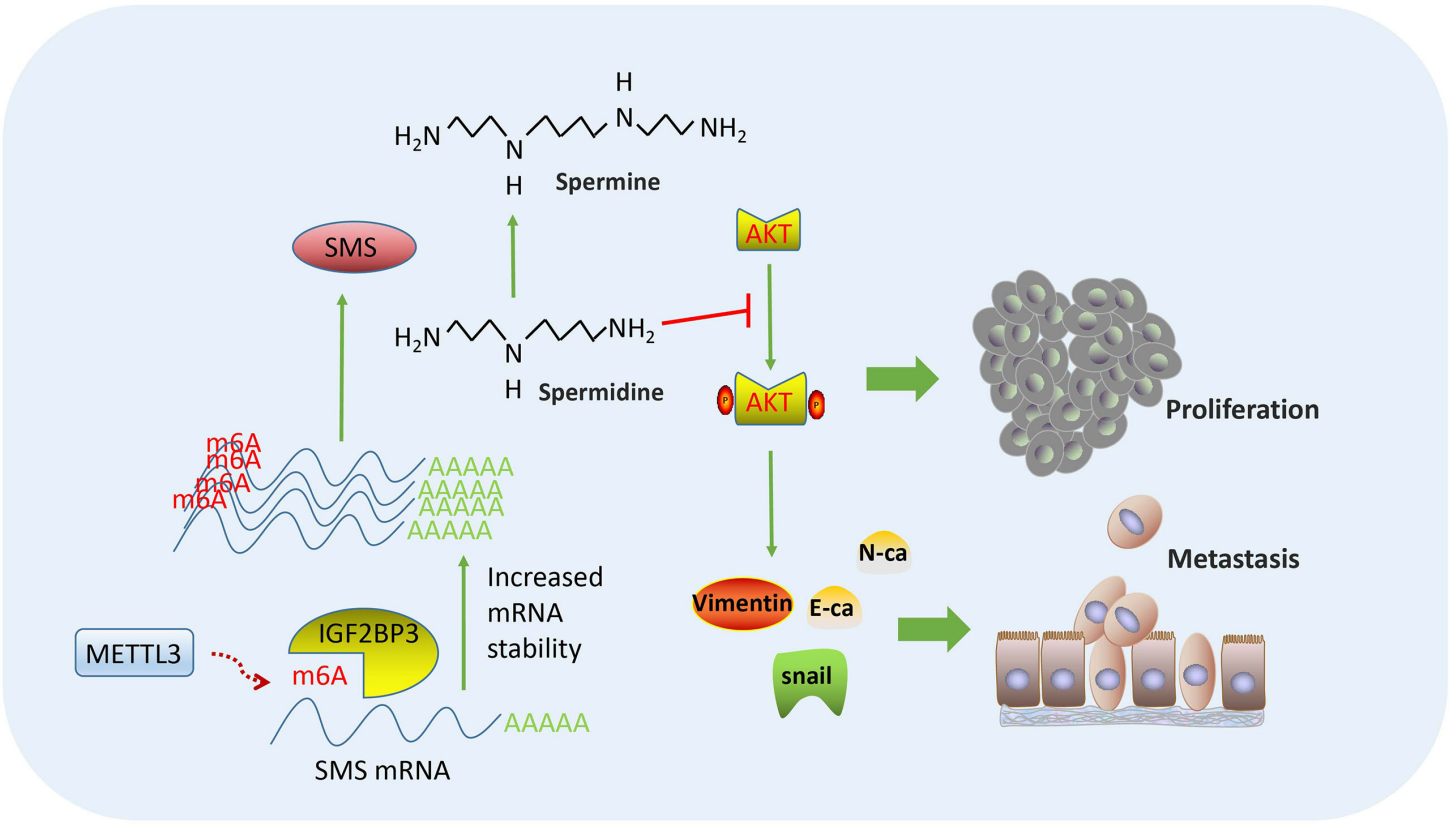
Flowchart of our experiments: METTL3-IGF2BP3 axis promotes pancreatic cancer progression by mediating *SMS* m6A modification.

In summary, our findings verified the effects of *SMS* on the proliferation, migration, and invasion of pancreatic cancer *in vitro* and *in vivo*. Moreover, overexpression of *SMS* could change the spermidine/spermine levels and further regulated the phosphorylation process of AKT and the state of PI3K-AKT/EMT signaling pathways. Finally, regarding the SMS regulation mechanism, we verified that the METTL3-IGF2BP3-axis could promote the migration and invasion of pancreatic cancer and that *METTL3* and *IGF2BP3* further increased the stability of *SMS* mRNA by modifying *SMS* mRNA by m6A. This led to increased *SMS* protein expression, the regulation of which relied on m6A modification of its mRNA (**Figure 8**).

## Conclusion

SMS promotes pancreatic cancer progression *in vitro* and *in vivo*. Overexpression of SMS could change the spermidine/spermine levels and further regulate the phosphorylation of AKT and the state of PI3K-AKT/EMT signaling pathways.The METTL3-IGF2BP3-axis could increase the stability of SMS mRNA in a m6A-dependent manner.

## Data availability statement

The datasets presented in this study can be found in online repositories. The names of the repository/repositories and accession number(s) can be found below: https://www.ncbi.nlm.nih.gov/geo/, GSE15471, GSE16515, GSE71989 and GSE22780. The data used to support the findings of this study are available from the corresponding author upon request.

## Ethics statement

The animal study was reviewed and approved by The animal experiments were approved by Animal Welfare Committee of Fujian Medical University (Fuzhou, China).

## Author contributions

Conception and design: ZG, SW. Development of methodology: ZG, XZ, CL, YH, YZ, HG, ZZ. Acquisition of data (acquired and managed patients, provided facilities, etc.): ZG, XZ, CL, YH, YZ, HG, ZZ. Analysis and interpretation of data (e.g., statistical analysis, biostatistics, computational analysis): ZG, XZ, CL. Writing, review, and/or revision of the manuscript: ZG, XZ, CL, SW. Study supervision: SW. All authors contributed to the article and approved the submitted version.

## Acknowledgments

We would like to thank Prof. Xu Lin for his help with providing experimental instruments and platforms. Prof. Xinjian Lin for his help for editing the manuscript. Dr. Kunqi Chen for his help with bioinformatics analysis. Dr. Yun He, Dr. Wenjun Liao for their help with animal experiment. Dr. Jiajian Shi for his help with Immunohistochemistry.

## Conflict of interest

The authors declare that the research was conducted in the absence of any commercial or financial relationships that could be construed as a potential conflict of interest.

## Publisher’s note

All claims expressed in this article are solely those of the authors and do not necessarily represent those of their affiliated organizations, or those of the publisher, the editors and the reviewers. Any product that may be evaluated in this article, or claim that may be made by its manufacturer, is not guaranteed or endorsed by the publisher.

## Abbreviations

METTL3, methyltransferase like 3; IGF2BP3, Insulin like growth factor 2 mRNA binding protein 3; AKT, Serine/threonine kinase; ME-RIP, Methylated RNA immunoprecipitation;M6A,N6- methyladenosine;SMS, Spermine Synthase; GEO, Gene Expression Omnibus; H&E, hematoxylin and eosin; IHC, immunohistochemistry; LC/MS, Liquid chromatography-tandem mass spectrometry; mTOR, mechanistic target of rapamycin; NC, negative control; ATCD, Actinomycin D; qRT-PCR, quantitative real-time polymerase chain reaction; TCGA, The Cancer Genome Atlas; GEPIA, Gene Expression Profifiling Interactive Analysis.

## References

[1] Drouillard A Manfredi S Lepage C Bouvier AM. Epidemiology of pancreatic cancer. Bull Du Cancer (2018) 105(1):63–9. doi: 10.1016/j.bulcan.2017.11.004 29273548

[2] Au M Emeto T Power J Vangaveti V Lai HJB. Emerging therapeutic potential of nanoparticles in pancreatic cancer: A systematic review of clinical trials. Biomedecines (2016) 4(3):20. doi: 10.3390/biomedicines4030020 PMC5344258 28536387

[3] Neoptolemos J Stocken D Bassi C Ghaneh P Cunningham D Goldstein D. Adjuvant chemotherapy with fluorouracil plus folinic acid vs gemcitabine following pancreatic cancer resection: a randomized controlled trial. JAMA (2010) 304(10):1073–81. doi: 10.1001/jama.2010.1275 20823433

[4] Lan Q Liu PY Haase J Bell JL Huttelmaier S Liu T. The critical role of RNA m(6)A methylation in cancer. Cancer Res (2019) 79(7):1285–92. doi: 10.1158/0008-5472.CAN-18-2965 30894375

[5] Wang T Kong S Tao M Ju S. The potential role of RNA N6-methyladenosine in cancer progression. Mol Cancer (2020) 19(1):88. doi: 10.1186/s12943-020-01204-7 32398132 PMC7216508

[6] He LE Li HY Wu AQ Peng YL Shu G Yin G. Functions of N6-methyladenosine and its role in cancer. Mol Cancer (2019) 18(1):176. doi: 10.1186/s12943-019-1109-9 31801551 PMC6892141

[7] Tan FH Zhao MY Xiong F Wang YM Zhang SS Gong ZJ. N6-methyladenosine-dependent signalling in cancer progression and insights into cancer therapies. J Exp Clin Cancer Res (2021) 40(1):146. doi: 10.1186/s13046-021-01952-4 33926508 PMC8082653

[8] Guo YD Wang RL Li JQ Song Y Min J Zhao T. Comprehensive analysis of m6A RNA methylation regulators and the immune microenvironment to aid immunotherapy in pancreatic cancer. Front Immunol (2021) 12:769425. doi: 10.3389/fimmu.2021.769425 34804059 PMC8602908

[9] Xia TF Wu XQ Cao M Zhang PB Shi GD Zhang JJ. The RNA m6A methyltransferase METTL3 promotes pancreatic cancer cell proliferation and invasion. Pathol Res Pract (2019) 215(11):152666. doi: 10.1016/j.prp.2019.152666 31606241

[10] Zeng J Zhang HY Tan YG Wang Z Li YW Yang XH. m6A demethylase RD suppresses pancreatic cancer tumorigenesis by demethylating PJA2 and inhibiting wnt signaling. Mol Therapy-Nucleic Acids (2021) 25:277–92. doi: 10.1016/j.omtn.2021.06.005 PMC8385122 34484859

[11] Chen Y Zhao Y Chen J Peng C Zhang Y Tong R. ALKBH5 suppresses malignancy of hepatocellular carcinoma *via* m(6)A-guided epigenetic inhibition of LYPD1. Mol Cancer (2020) 19(1):123. doi: 10.1186/s12943-020-01239-w 32772918 PMC7416417

[12] Wu Y Chang N Zhang Y Zhang X Xu L Che Y. METTL3-mediated m(6)A mRNA modification of FBXW7 suppresses lung adenocarcinoma. J Exp Clin Cancer Res (2021) 40(1):90. doi: 10.1186/s13046-021-01880-3 33676554 PMC7936500

[13] Arruabarrena-Aristorena A Zabala-Letona A Carracedo A. Oil for the cancer engine: The cross-talk between oncogenic signaling and polyamine metabolism. Sci Adv (2018) 4:eaar2606. 29376126 10.1126/sciadv.aar2606 PMC5783676

[14] Wang X Ying W Dunlap KA Lin G Satterfield MC Burghardt RC. Arginine decarboxylase and agmatinase: an alternative pathway for *de novo* biosynthesis of polyamines for development of mammalian conceptuses. Biol Reprod (2014) 90(4):84. doi: 10.1095/biolreprod.113.114637 24648395

[15] Russell DH Gerner EW. The relationship between polyamine accumulation and DNA replication in synchronized Chinese hamster ovary cells after heat shock. Cancer Res (1977) 37:482–9. 832272

[16] Massaro C Thomas J Phanstiel Iv O. Investigation of polyamine metabolism and homeostasis in pancreatic cancers. Med Sci (Basel) (2017) 5(4):32. doi: 10.3390/medsci5040032 PMC5753661 29215586

[17] Pietrocola F Lachkar S Enot DP Niso-Santano M Bravo-San Pedro JM Sica V. Spermidine induces autophagy by inhibiting the acetyltransferase EP300. Cell Death Differ (2015) 22(3):509–16. doi: 10.1038/cdd.2014.215 PMC4326581 25526088

[18] Phanstiel O. An overview of polyamine metabolism in pancreatic ductal adenocarcinoma. Int J Cancer (2018) 142(10):1968–76. doi: 10.1002/ijc.31155 29134652

[19] Ogata K Nishimoto N Uhlinger DJ Igarashi K Takeshita M Tamura M. Spermine suppresses the activation of human neutrophil NADPH oxidase in cell-free and semi-recombinant systems. Biochem J (1996) 313(Pt 2):549–54. doi: 10.1042/bj3130549 PMC1216942 8573091

[20] Madeo F Eisenberg T Pietrocola F Kroemer G. Spermidine in health and disease. Science (2018) 359(6374):eaan2788. doi: 10.1126/science.aan2788 29371440

[21] Guo Y Ye Q Deng P Cao Y He D Zhou Z. Spermine synthase and MYC cooperate to maintain colorectal cancer cell survival by repressing bim expression. Nat Commun (2020) 11(1):3243. doi: 10.1038/s41467-020-17067-x 32591507 PMC7320137

[22] Wang H Zhang K Liu J Yang J Tian Y Yang C. Curcumin regulates cancer progression: Focus on ncRNAs and molecular signaling pathways. Front Oncol (2021) 11:660712. doi: 10.3389/fonc.2021.660712 33912467 PMC8072122

[23] Polivka J Jr. Janku F. Molecular targets for cancer therapy in the PI3K/AKT/mTOR pathway. Pharmacol Ther (2014) 142(2):164–75. doi: 10.1016/j.pharmthera.2013.12.004 24333502

[24] Karimi Roshan M Soltani A Soleimani A Rezaie Kahkhaie K Afshari AR Soukhtanloo M. Role of AKT and mTOR signaling pathways in the induction of epithelial-mesenchymal transition (EMT) process. Biochimie (2019) 165:229–34. doi: 10.1016/j.biochi.2019.08.003 31401189

[25] Nakkina SP Gitto SB Pandey V Parikh JG Geerts D Maurer HC. Differential expression of polyamine pathways in human pancreatic tumor progression and effects of polyamine blockade on tumor microenvironment. Cancers (Basel) (2021) 13(24):6391. doi: 10.3390/cancers13246391 34945011 PMC8699198

[26] Wang J Ye Q Cao Y Guo Y Huang X Mi W. Snail determines the therapeutic response to mTOR kinase inhibitors by transcriptional repression of 4E-BP1. Nat Commun (2017) 8(1):2207. doi: 10.1038/s41467-017-02243-3 29263324 PMC5738350

[27] Ge H Liang C Li Z An D Ren S Yue C. DcR3 induces proliferation, migration, invasion, and EMT in gastric cancer cells *via* the PI3K/AKT/GSK-3beta/beta-catenin signaling pathway. Onco Targets Ther (2018) 11:4177–87. doi: 10.2147/OTT.S172713 PMC6056154 30050309

[28] Byun JA Lee SH Jung BH Choi MH Moon MH Chung BC. Analysis of polyamines as carbamoyl derivatives in urine and serum by liquid chromatography-tandem mass spectrometry. BioMed Chromatogr (2008) 22(1):73–80. doi: 10.1002/bmc.898 17668437

[29] Tantak MP Sekhar V Tao X Zhai RG Phanstiel O. Development of a redox-sensitive spermine prodrug for the potential treatment of Snyder Robinson syndrome. J Med Chem (2021) 64(21):15593–607. doi: 10.1021/acs.jmedchem.1c00419 PMC9115777 34695351

[30] Wu H Min J Zeng H McCloskey D Ikeguchi Y Loppnau P. Crystal structure of human spermine synthase: implications of substrate binding and catalytic mechanism. J Biol Chem (2008) 283(23):16135–46. doi: 10.1074/jbc.M710323200 PMC3259631 18367445

[31] Stathis A Moore MJ. Advanced pancreatic carcinoma: current treatment and future challenges. Nat Rev Clin Oncol (2010) 7(3):163–72. doi: 10.1038/nrclinonc.2009.236 20101258

[32] Miranda-Filho A Bray F Charvat H Rajaraman S Soerjomataram I. The world cancer patient population (WCPP): An updated standard for international comparisons of population-based survival. Cancer Epidemiol (2020) 69:101802. doi: 10.1016/j.canep.2020.101802 32942139 PMC7768180

[33] Li LI Li J J. NRAO Li M Bass BL Wang JY. Inhibition of polyamine synthesis induces p53 gene expression but not apoptosis. Am J Physiol (1999) 276(4):C946–54. doi: 10.1152/ajpcell.1999.276.4.C946 10199827

[34] Kaul D Wu CL Adkins CB Jordan KW Defeo EM Habbel P. Assessing prostate cancer growth with mRNA of spermine metabolic enzymes. Cancer Biol Ther (2010) 9(9):736–42. doi: 10.4161/cbt.9.9.11549 20215859

[35] Chrisam M Pirozzi M Castagnaro S Blaauw B Polishchuck R Cecconi F. Reactivation of autophagy by spermidine ameliorates the myopathic defects of collagen VI-null mice. Autophagy (2015) 11(12):2142–52. doi: 10.1080/15548627.2015.1108508 PMC4835186 26565691

[36] Campagna MP Xavier A Lechner-Scott J Maltby V Scott RJ Butzkueven H. Epigenome-wide association studies: current knowledge, strategies and recommendations. Clin Epigenet (2021) 13(1):214. doi: 10.1186/s13148-021-01200-8 PMC8645110 34863305

[37] Dominissini D Moshitch-Moshkovitz S Schwartz S Salmon-Divon M Ungar L Osenberg S. Topology of the human and mouse m6A RNA methylomes revealed by m6A-seq. Nature (2012) 485(7397):201–6. doi: 10.1038/nature11112 22575960

[38] Guo W Zhang C Feng P Li M Wang X Xia Y. M6A methylation of DEGS2, a key ceramide-synthesizing enzyme, is involved in colorectal cancer progression through ceramide synthesis. Oncogene (2021) 40(40):5913–24. doi: 10.1038/s41388-021-01987-z PMC8497269 34363020

[39] Ma L Lin Y Sun S Xu J Yu T Chen W. KIAA1429 is a potential prognostic marker in colorectal cancer by promoting the proliferation *via* downregulating WEE1 expression in an m6A-independent manner. Oncogene. (2022) 41(5):692–703. doi: 10.1038/s41388-021-02066-z 34819634

[40] Fang R Chen X Zhang S Shi H Ye Y Shi H. EGFR/SRC/ERK-stabilized YTHDF2 promotes cholesterol dysregulation and invasive growth of glioblastoma. Nat Commun (2021) 12(1):177. doi: 10.1038/s41467-020-20379-7 33420027 PMC7794382

[41] Han H Yang C Zhang S Cheng M Guo S Zhu Y. METTL3-mediated m(6)A mRNA modification promotes esophageal cancer initiation and progression *via* notch signaling pathway. Mol Ther Nucleic Acids (2021) 26:333–46. doi: 10.1016/j.omtn.2021.07.007 PMC8416973 34513313

[42] Chen S Yang C Wang ZW Hu JF Pan JJ Liao CY. CLK1/SRSF5 pathway induces aberrant exon skipping of METTL14 and cyclin L2 and promotes growth and metastasis of pancreatic cancer. J Hematol Oncol (2021) 14(1):60. doi: 10.1186/s13045-021-01072-8 33849617 PMC8045197

