# Supplementary material for: METTL3-IGF2BP3-axis mediates the proliferation and migration of pancreatic cancer by regulating spermine synthase m6A modification
Source: Front Oncol. 2022 Oct 6;12:962204. doi: 10.3389/fonc.2022.962204 (PMC9582246; doi:10.3389/fonc.2022.962204)
Supplement: Supplementary file 1 [file DataSheet_1.docx]

Supplementary Material

## Supplementary Figures

**Supplementary Figure 1.** **(A)** Expression level of m6A readers in pancreatic tumor and adjacent normal tissues from TCGA database were analyzed. Kaplan-Meier analyses of *IGF2BP3* high expression and low expression were analyzed from the TCGA database. **(B and C)** Wound-healing assays show that interference with *METTL3* inhibits PANC1 and Mia-Paca2 cell migration. Representative images and quantification of wound closure are presented. *, P < 0.05; **, P < 0.01 by t-test.

**
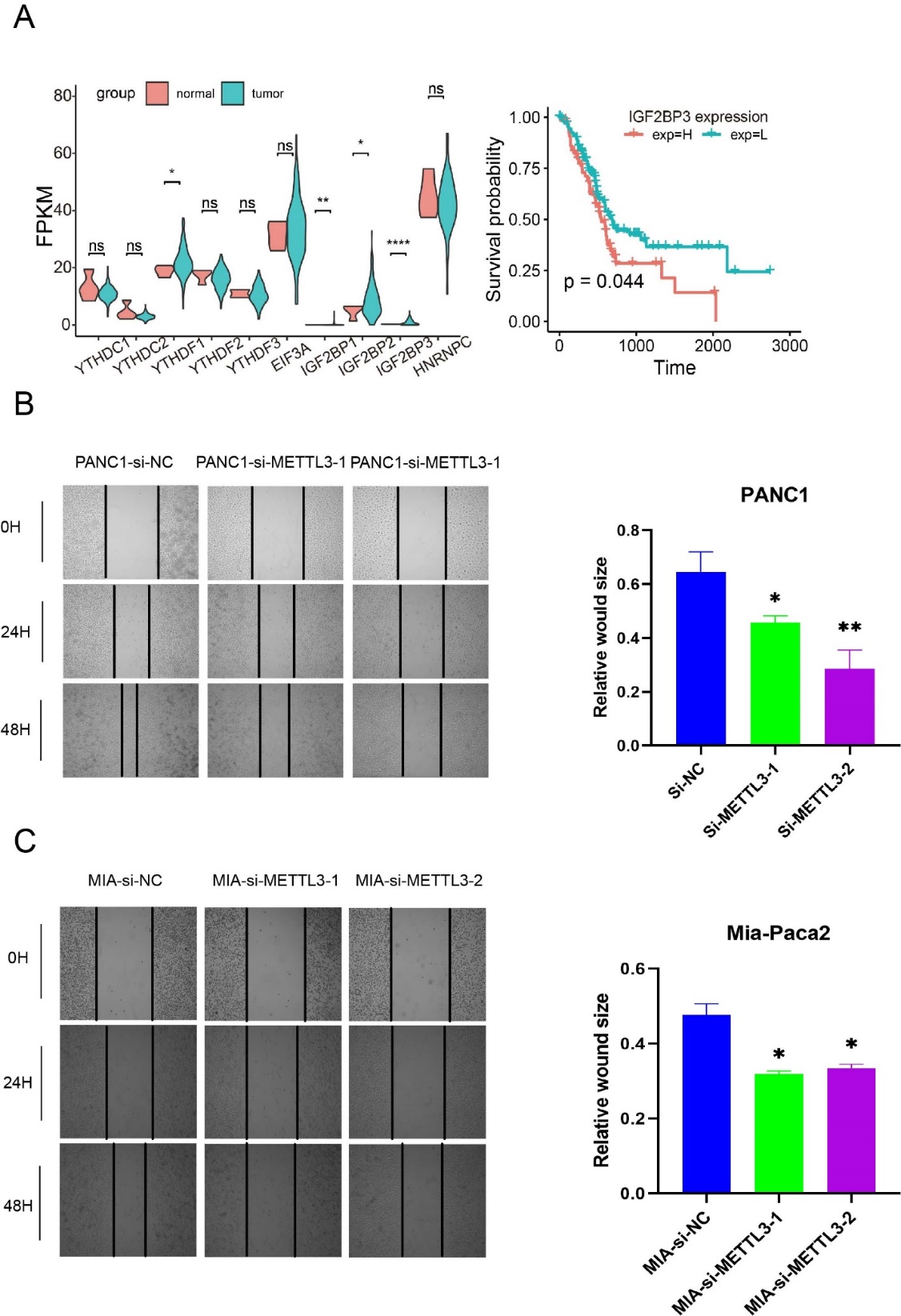
**

**Supplementary Figure 2. (A, B)**The protein expressions of ERK, p-ERK, mTOR and p-mTOR were analyzed in SMS-OE or SMS-knockdown Mia-Paca2 and PANC1 cells.


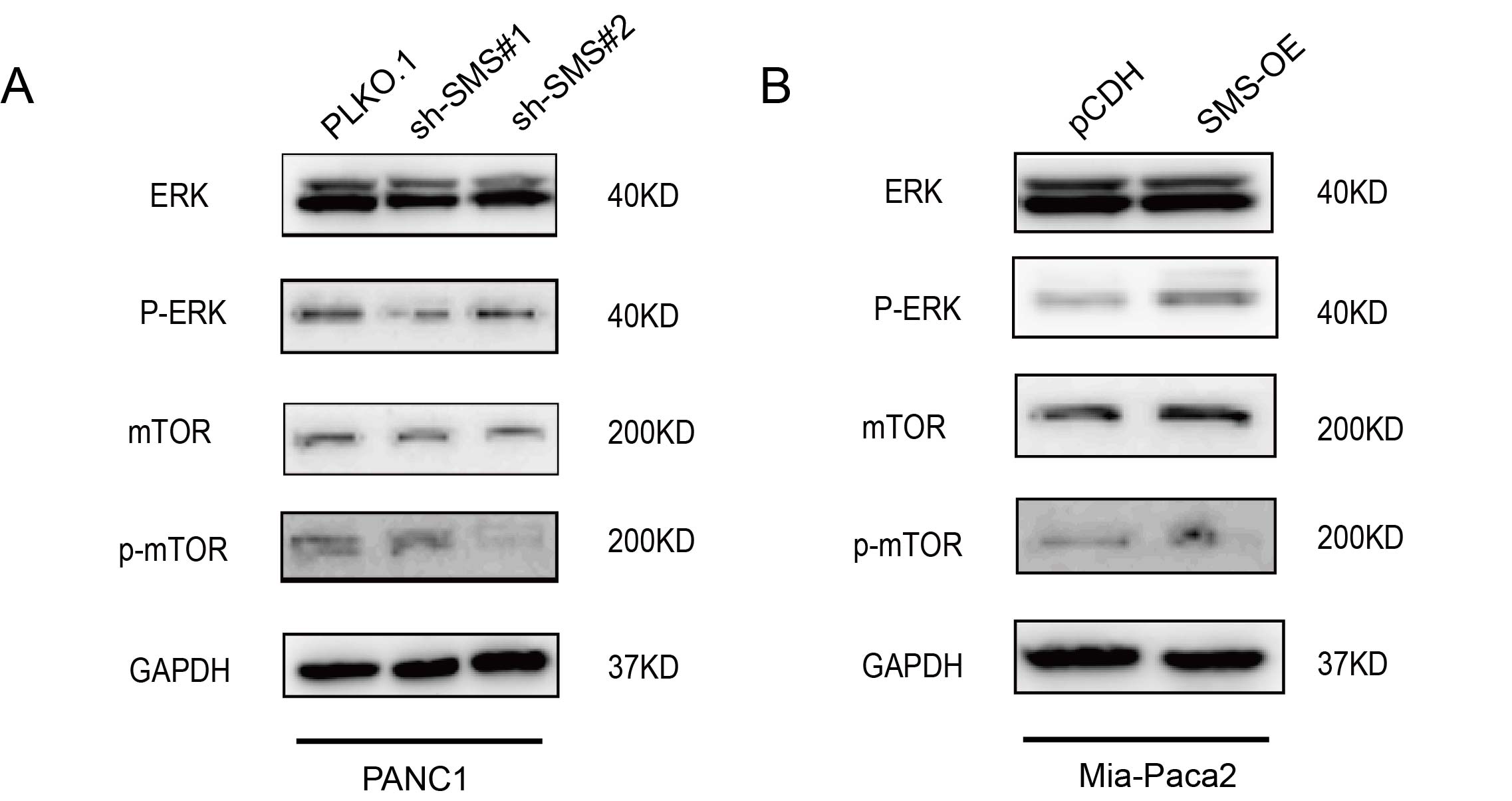


## Supplementary Tables

**Supplementary Table S1:** Relationship between *SMS* expression and clinicopathological characteristics of pancreatic cancer patient.

|  | N=59 | High SMS | Low SMS | χ^2^ | *p* |
| --- | --- | --- | --- | --- | --- |
| Gender |  |  |  |  |  |
| Male | 31 | 20 | 11 | 0.091 | 0.763 |
| Female | 28 | 17 | 11 |  |  |
| Age |  |  |  |  |  |
| ≥60 | 23 | 14 | 9 | 0.055 | 0.815 |
| ＜60 | 36 | 23 | 13 |  |  |
| Tumor size |  |  |  |  |  |
| ＞4cm | 15 | 8 | 7 | 0.757 | 0.384 |
| ≤4cm | 44 | 29 | 15 |  |  |
| Tumor location  in pancreas |  |  |  |  |  |
| Head | 41 | 24 | 17 | 1.002 | 0.317 |
| Body / tail | 18 | 13 | 5 |  |  |
| Differentiation |  |  |  |  |  |
| Low | 8 | 5 | 3 | 0.000 | 0.989 |
| Middle/high | 51 | 32 | 19 |  |  |
| Lymphatic metastasis |  |  |  |  |  |
| Yes | 42 | 30 | 12 | 4.736 | 0.03* |
| No | 17 | 7 | 10 |  |  |
| TNM stage |  |  |  |  |  |
| Ⅰ/Ⅱ | 31 | 14 | 17 | 8.604 | 0.003* |
| Ⅲ/Ⅳ | 28 | 23 | 5 |  |  |

**p*＜0.05 was considered significant

**Supplementary Table S2:** si-RNA base sequence

| Name | Forward (5’→3’) | Reverse (5’→3’) |
| --- | --- | --- |
| Si-METTL3-1 | GGUUGGUGUCAAAGGAAAUTT | AUUUCCUUUGACACCAACCTT |
| Si-METTL3-2 | GGUGACUGCUCUUUCCUUATT | UAAGGAAAGAGCAGUCACCTT |
| Si-METTL14-1 | GGAUGAAGGAGAGACAGAUTT | AUCUGUCUCUCCUUCAUCCTT |
| Si-METTL14-2 | GCAGCACCUCGAUCAUUUATT | UAAAUGAUCGAGGUGCUGCTT |
| Si-IGF2BP3-1 | GGCUCAGGGAAGAAUUUAUTT | AUAAAUUCUUCCCUGAGCCTT |
| Si-IGF2BP3-2 | GCUGCUGAGAAGUCGAUUATT | UAAUCGACUUCUCAGCAGCTT |

**Supplementary Table S3:** q-PCR primers.

| Name | Forward (5’→3’) | Reverse (5’→3’) |
| --- | --- | --- |
| METTL3 | CTGCAACGCATCATTCGGAC | AGACCCTGGTTGAAGCCTTG |
| IGF2BP3 | GCTCATATCAGAGTGCCATCC | TGGTCATTCTCATCAGGTGTCT |
| SMS | AAGGAGACTGCTATCAGGTTCTA | ATGTGGAATCTTCTTCTGGAGAC |
| GAPDH | GGTGTGAACCATGAGAAGTATGA | GAGTCCTTCCACGATACCAAAG |
